# Fear for external cephalic version and depression: predictors of successful external cephalic version for breech presentation at term?

**DOI:** 10.1186/1471-2393-14-101

**Published:** 2014-03-12

**Authors:** Emily Ciliacus, Marieke van der Zalm, Sophie E Truijens, Tom H Hasaart, Victor J Pop, Simone M Kuppens

**Affiliations:** 1 Department of Obstetrics and Gynecology, Catharina Hospital, Eindhoven, the Netherlands; 2 Department of Medical and Clinical Psychology, Tilburg University, Tilburg, the Netherlands

**Keywords:** External cephalic version, Breech presentation, EDS, Depression, Psychological predictors

## Abstract

**Background:**

Objective was to determine whether fear for external cephalic version (ECV) and depression are associated with the success rate of ECV in women with a breech presentation at term.

**Methods:**

Prospective study conducted in the Catharina Hospital Eindhoven between October 2007 and May 2012. Participants fulfilled The Edinburgh Depression Scale (EDS) questionnaire and expressed their degree of fear on a visual analogue scale from one to ten before ECV. Obstetric factors were evaluated as well. Primary outcome was the relation between psychological factors (fear for ECV and depression EDS scores) and ECV success rate. Secondary outcome was a possible relation between fear for ECV and increased abdominal muscle tension.

**Results:**

The overall success rate was 55% and was significantly lower (p < 0.001) in nulliparous women (44.3%) compared with parous women (78.0%). Fear for ECV and depression EDS-scores were not related with ECV success rate. Parity, placental location, BMI and engagement of the fetal breech were obstetric factors associated with ECV outcome. There was no relation between fear for ECV and abdominal muscle tone.

**Conclusion:**

Fear for ECV and depression were not related with ECV success rate in this study. Engagement of the fetal breech was the most important factor associated with a successful ECV.

**Trial registration:**

EBIS: The Eindhoven Breech Intervention Study, NCT00516555.

## Background

External cephalic version (ECV) is the best method to reduce the number of breech positions at term and is recommended by the guidelines of the ACOG and RCOG [1,2]. ECV has become more popular in the past 10 years due increasing demand for the reduction of caesarean sections (CS), a strong safety record, and high success rates of ECV of up to 80% [3].

Factors predicting the outcome of ECV have been identified, but until now prediction models and scoring systems have not been satisfactory [4]. Previous studies show that obstetric factors significantly associated with ECV success rate are: parity, engagement of the fetal presenting part, placental location, type of breech and amount of amniotic fluid [5-7]. Among these factors, parity seems to be the most important factor to predict ECV success rate, with nulliparity being a negative predictor.

The success rate of ECV is not only related to physical, obstetric and fetal factors but may be influenced by other factors as well, such as practitioner skills, maternal attitude, expectations and stress [8]. Fear is an important factor with approximately 25% of the women refusing ECV because of fear of pain and fetal distress [9]. We hypothesize that fear for ECV can predispose to failure of the attempt, due to increased abdominal muscle tension and discontinuation of the attempt on patient’s request. Moreover, previous studies showed that antepartum depression may have deleterious effects on peripartum maternal and neonatal outcomes [10]. We hypothesize that maternal mood status might also have an effect on ECV success rate. Our hypothesis is that women who are more depressed tend to have more negative expectations towards ECV. This may lead to less cooperation and unintended increase of abdominal muscle tension and thereby to a lower success rate.

Very little is known about the effect of fear and maternal mood status before ECV on the outcome of ECV. As far as we know now, this is one of the first studies reporting the relation between psychological factors and ECV success rate.

## Methods

### Study design

A prospective observational study was conducted between October 2007 and May 2012 in the Catharina Hospital in Eindhoven, The Netherlands. The study was approved by the Medical Ethical Committee of the Catharina Hospital. Informed consent was obtained from all participants.

### ECV intervention

The Obstetric department of the Catharina Hospital has extensive experience in external cephalic version. All ECV procedures during the study period were performed by the same two operators, one trained obstetric gynecologist and one trained midwife. The hands of one staff member concentrated on the breech, while the hands of the other staff member concentrated on the fetal head, with manipulation being consecutive rather than simultaneous. ‘Forward somersault’ was the preferred method to achieve cephalic position, and a ‘backward flip’ was an alternative strategy for nulliparous women with a frank breech presentation [11].

Before and after each ECV procedure the fetus was monitored by cardiotocography (CTG). Fetal ultrasound was used before ECV to determine fetal position, estimated fetal weight, placental location and amniotic fluid index (AFI). A tocolytic agent (Tractocile, 6.75 mg intravenously) was used in all ECV attempts.

### Participants

Pregnant women who underwent ECV for breech presentation between October 2007 and May 2012 were included. Exclusion criteria were maternal age under 18 years, gestational age less than 35 weeks, a history of caesarean section (CS), no mastery of the Dutch language and contraindications to ECV. Because higher TSH levels may increase the risk of ECV failure we also excluded women with maternal thyroid disease and other maternal autoimmune disease [11].

### Assessments

Assessment of depressive symptoms was performed by means of the Dutch version of the Edinburgh Depression Scale (EDS). This 10 item questionnaire is designed to screen for symptoms of emotional distress, in the past seven days [12] EDS had originally been developed as EPDS to screen for emotional stress in the post partum period. However EDS has now been validated for screening during pregnancy. A total EDS-score is determined by adding the scores for each of the 10 items and ranges from 0 to 30, with higher scores indicating more symptoms of depression. A cut off point of 10 or higher is used to define clinical relevant signs of depression in third trimester of pregnancy [12]. Before ECV procedure, all participants were asked to fulfill this questionnaire. Fear for the procedure was measured by rating the fear for ECV prior to the version on a 10-points visual analog scale (VAS) from one (no fear at all) to ten (extremely fearful).

Before ECV, several obstetric factors were documented by the operators: gestational age (weeks and days), type of breech (frank versus non-frank), placental location (anterior versus non-anterior), AFI (≤10 or > 10) abdominal muscles tone (strong versus weak or normal), uterine tone (intense versus relaxed or normal), engagement of the fetal breech (above or in pelvic inlet), palpability of the fetal head (yes or no) and estimated fetal weight (EFW by ultrasound). Abdominal muscle tone, uterine tone and engagement of the fetal breech were subjective assessments measured by the obstetric gynaecologist and the midwife who performed the ECV.

Primary outcome was the possible relation between psychological factors (fear for ECV and depression) and the ECV success rate. Secondary outcome was a possible relation between fear for ECV and increased abdominal muscle tension.

### Data analysis and processing

Statistical analysis was carried out using the Statistical Package for Social Sciences for Windows 19.0 (SPSS). The mean (SD), median (range) or numbers of patients were processed for each baseline characteristic and were shown for nulliparous and parous women separately. Simple logistic regression, with ECV outcome as dependent variable and psychological factors as independent variables, was used to select variables significantly associated with ECV success rate. Subsequently, we evaluated these associations (all variables with P < 0.1) in a multiple logistic regression model (OR 95% CI), taking into account confounders such as the above named obstetric variables. A *p*-value <0.05 was considered statistically significant.

## Results

During the study period 253 women were included for the study based on the inclusion- and exclusion criteria. Results of four of these women were not analyzed because of missing data, leaving 249 women, 167 nulliparous women and 82 parous women, for the analyses of the baseline characteristics (Table 1).

**Table 1:** Characteristics of 249 women who underwent ECV between October 2007 and May 2012

	**All women (N = 249)**		**Nulliparous women (N = 167)**		**Parous women (N = 82)**		**P-value**
	**N (%)**	**Mean (SD)/Median (min-max)**	**N (%)**	**Mean (SD)/Median (min-max)**	**N (%)**	**Mean (SD)/Median (min-max)**	
**Demographic features**							
Maternal age (years)		31.43 (4.22)		30.95 (4.36)		32.41 (3.75)	**0.010**^**1**^
*BMI*		22.69 (17.4-47.3)		22.60 (17.7-38.9)		22.70 (17.4-47.3)	0.687^3^
<30	226 (90.8)		153 (91.6)		73 (89.0)		
≥30	23 (9.2)		14 (8.4)		9 (11.0)		
Any tobacco use	16 (6.4)		11 (6.6)		5 (6.1)		0.416^2^
Any alcohol use	13 (5.2)		9 (5.4)		4 (4.9)		0.457^2^
**Obstetrical features**							
Gestational age at ECV		36.07 (0.80)		35.98 (0.74)		36.24 (0.89)	**0.017**^**1**^
< 37	222 (89.2)		151 (90.4)		71 (86.6)		
≥ 37	27 (10.8)		16 (9.6)		11 (13.4)		
*Type of breech*							0.055^2^
Non-frank	80 (32.1)		47 (28.1)		33 (40.2)		
Frank	169 (67.9)		120 (71.9)		49 (59.8)		
*Placenta location*							0.793^2^
Posterior/lateral	159 (64.1)		108 (64.7)		51 (62.2)		
Anterior	89 (35.9)		59 (35.3)		30 (36.6)		
*AFI*							0.117^2^
>10	99 (39.9)		61 (36.5)		38 (46.9)		
≤10	149 (60.1)		106 (63.5)		43 (53.1)		
*Tonus abdominal muscles*							**0.008**^**2**^
Weak/normal	205 (82.3)		130 (77.8)		75 (91.5)		
Strong	44 (17.7)		37 (22.2)		7 (8.5)		
*Tonus uterus*							0.033^2^
Relaxed/normal	189 (75.9)		120 (71.9)		69 (84.1)		
Intense	60 (24.1)		47 (28.1)		13 (15.9)		
*Engagement*							**<0.001**^**2**^
Breech above pelvic inlet	118 (47.6)		65 (39.2)		53 (64.6)		
Breech in pelvic inlet	130 (52.4)		101 (60.8)		29 (35.4)		
*Head palpable*							0.629^2^
Yes	231 (92.8)	3.73 (2.86)	154 (92.2)	4.09 (2.68)	77 (93.9)	3.00 (3.10)	
No	18 (7.2)	2626.22 (325.62)	13 (7.8)	2599.83 (319.76)	5 (6.1)	2679.63 (332.77)	
Duration ECV (min)							**0.005**^**1**^
EFW (gram)							0.069^1^
**Psychosocial features**							
Degree of fear before ECV		5.31 (2.24)		5.35 (2.22)		5.22 (2.28)	0.659^1^
Total EDS- score		4.28 (3.96)		4.07 (4.07)		4.71 (3.71)	0.231^1^
EDS >10		18 (7.2)		14 (8.4)		4 (4.9)	0.315^2^
**Outcome**							
Cephalic presentation after ECV		138 (55.4)		74 (44.3)		64 (78.0)	**<0.001**^**2**^

^1^=t-test^1^.

^2^=chi-square test^2^.

^3^=non-parametric test^3^.

Bold numbers are statistically significant numbers.

The overall ECV success rate was 55% and was significantly lower (p < 0.001) in nulliparous women (44%) compared with parous women (78%). Gestational age at ECV ranged from 35 weeks to 39 weeks and 6 days (mean 36 weeks) and was significantly higher in parous women (p = 0.017). In total 222 women underwent ECV before 37 weeks of pregnancy (151 nulliparous women and 71 parous women). Twenty-seven women underwent ECV after 37 weeks (16 nulliparous women and 11 parous women). Nulliparous women differed from parous women in that they were younger (p = <0.01) and that the duration of the ECV attempt was longer (p =0.005). They more often had a frank breech (p = 0.055), engaged breech (p = <0.001), strong abdominal muscles (p = 0.008) and intense uterine tone (p = 0.03). Psychosocial features (fear of ECV and EDS-scores) were not significantly different between nulliparous and parous women (Table 1). The Pearson correlation between fear for ECV and EDS-score was 0.387 and significant at the 0.01 level.

Simple logistic regression, with ECV success rate as a primary outcome, showed that BMI, parity, type of breech, placental location, AFI, abdominal muscle tone, uterine tone and engagement of the fetal breech were significantly associated with ECV success rate. Fear for ECV and EDS-score (depression) were not related with ECV success rate (Table 2). After correction for confounders with multi logistic regression only multi parity (OR 3.56, 95% CI: 1.73-7.32), non-anterior placental location (OR 2.77, 95% CI: 1.40-5.47), lower BMI (OR 0.90, 95% CI: 0.84-0.98) and non-engagement of the fetal breech (OR 6.79, 95% CI: 3.42-13.51) were significant positive predictors of ECV success rate (Table 2).

**Table 2:** logistic regression of 249 women who underwent ECV, outcome successful ECV

	**Simple logistic regression**			**Multiple logistic regression**		
	**OR**	**[95% BI]**	**P-value**	**OR**	**[95% CI]**	**P-value**
**Demographic features**						
Maternal age (years)	1.06	[1.00-1.13]	0.056	1.02	[0.95-1.10]	0.626
BMI	0.93	[0.87-0.99]	**0.014**	0.90	[0.84-0.98]	**0.013**
**Obstetrical features**						
*Parity*						
Parous women	4.47	[2.44-8.19]	**<0.001**	3.56	[1.73-7.32]	**0.001**
Nulliparous women	1.00			1.00		
Gestational age at ECV	0.99	[0.72-1.35]	0.934			
*Type of breech*						
Non-frank	2.69	[1.52-4.76]	**0.001**	1.26	[0.60-2.68]	0.55
Frank	1.00			1.00		
*Placenta location*						
Posterior/lateral	2.38	[1.40-4.05]	**0.001**	2.77	[1.40-5.47]	**0.003**
Anterior	1.00			1.00		
*AFI*						
>10	2.54	[1.49-4.33]	**0.001**	1.79	[0.91-3.50]	0.091
≤10	1.00			1.00		
*Tonus of abdominal muscles*						
Weak/normal	2.29	[1.17-4.46]	**0.015**	1.41	[0.52-3.82]	0.497
Strong	1.00			1.00		
*Tonus of uterus*						
Relaxed/normal	2.28	[1.26-4.13]	**0.006**	1.22	[0.51-2.95]	0.653
Intense	1.00			1.00		
Engagement						
Breech above pelvic inlet	9.14	[5.06-16.52]	**<0.001**	6.79	[3.42-13.51]	**<0.001**
Breech in pelvic inlet	1.00			1.00		
Head palpable						
Yes	2.67	[0.97-7.35	0.058	0.90	[0.25-3.24]	0.875
No	1.00					
EFW (gram)	1.00	[1.00-1.00]	0.894			
**Psychosocial features**						
Degree of fear before ECV	0.92	[0.82-1.03]	0.160	0.94	[0.82-1.09]	0.430
EDS score before ECV	1.03	[0.97-1.10]	0.339			

Bold numbers are statistically significant numbers.

Additionally nulliparous women and parous women were analysed separately. Fear for ECV and EDS-scores were also not related with ECV success rate in these subgroups. Results are shown in an additional file (Additional file 1: Table S1 and Additional file 2: Table S2).

Secondary outcome was a possible relation between fear for ECV and increased abdominal muscle tension. When we analysed nulliparous en parous women together there was no relation between fear for ECV and abdominal muscle tone (p = 0.977). For nulliparous women en parous women separately there was also no correlation between fear for ECV and abdominal muscle tone (p = 0.612 respectively p = 0.347).

The most common complication after ECV was a non-reassuring fetal heart rate (2,4%); most often a transient bradycardia. Emergency CS was performed twice because of persistent problems with fetal heart rate. In both cases maternal and fetal outcome were good with Apgarscores of more than 7 after 5 minutes.

## Discussion

The total ECV success rate of 55% in this study is comparable to results reported in the literature (2) and was much higher for parous women compared to nulliparous women. In this study parity, BMI, placental location and engagement of the fetal breech were significantly related with the outcome of ECV. Psychological factors (fear for ECV and depression EDS-scores) were not related with ECV success rate in this study. Furthermore, there was no relation between fear for ECV and abdominal muscle tension.

In nulliparous women factors negatively influencing ECV success rate were more common, such as a more intense uterine and abdominal muscle tone and an engaged breech. Therefore, in nulliparous women manipulation of the fetus through the abdominal wall seemed to be more difficult and maybe less effective. The most important predictor for ECV success rate for both nulliparous women and parous women is a mechanic factor, namely engagement of the breech. This might be modified by performing ECV at an earlier stage in pregnancy, as has been described in the literature. However, the number of CS did not decrease despite higher ECV success rate [13]. We assume that nulliparous women might benefit most from early ECV intervention. There were no differences in EDS-scores or fear for ECV between nulliparous and multiparous women. The mean EDS-score was 4.28 (3.96 SD), which is comparable to EDS-scores of pregnant women in their third trimester described in the literature [12].

While our data support earlier observations that factors affecting ECV success include parity, BMI, placental location and engagement of the fetal breech [5-7], to our knowledge, this is the first study reporting on a possible relation between fear for ECV or depression before ECV and ECV outcome. Several studies describe the predictive value of obstetric parameters, but little is known about the effect of psychological factors on ECV success rate.

Primary outcome was a possible relation between fear for ECV or depression and ECV success rate. However, this relation was not found. Secondary outcome was a possible relation between fear for ECV and abdominal muscle tone. We hypothesized that fear for the procedure would lead to increased tension in the abdominal wall and hence to more difficult and less effective ECV. We found no relation between fear for ECV and abdominal muscle tension.

When we analysed nulliparous women and parous women separately (Additional file 1: Table S1 and Additional file 2: Table S2), fear for ECV and depression were again not related with the outcome of ECV.

There are many women who decline ECV because of fear. Since there seems to be no relation between fear and ECV success rate, we should encourage anxious women to undergo the procedure. Good explanation and with that better understanding of the procedure might minimize their fear. Intervention strategies to reduce anxiety, such as hypnosis or pain relief, might be helpful in these women [9,14].

In our study, transient fetal bradycardia after ECV occurred in 2. 4% of the cases. Two of the ECV attempts (0.8%) were followed by emergency CS due to persistent abnormal fetal heart rate patterns. In both cases maternal and fetal outcome was good. There was no ECV related fetal mortality in this study. A recent meta-analysis showed that transient abnormal cardiotocography patterns occur in 4.7% and emergency CS in 0.35% of all ECV-procedures [15]. The incidence of emergency CS in this cohort is slightly higher (2/249; 0.80%), most likely as a consequence of the relatively small cohort size. However, our overall incidence of emergency CS over the last years is 4 per 1000 (0.4%).

A limitation is this study is that we have no data of women who declined ECV because of fear for the procedure. Fear of pain and fetal distress has been described as an important factor with approximately 25% of the women refusing ECV because of that [9]. It might be possible that most anxious women were not part of this study. However, given the fact that there was a high correlation between fear for ECV and EDS and that the mean score of the EDS of the current study was comparable to a cohort of 1000 pregnant women of the general population [12], there is no reason to suggest that especially more anxious women did not participate into an ECV attempt.

Unfortunately we only looked at fear in a one-dimensional way and we did not distinguish between women’s fear for pain, fear for adverse outcome for the baby or fear for adverse outcome for herself. It is arguable that different causes for fear of ECV might have different effects on a woman’s willingness to accept ECV and ability to relax during the procedure.

Furthermore, some of the analyzed obstetric parameters (abdominal tone, uterine tone, engagement of fetal breech) were subjective assessments. Another limitation of the study is that only Dutch-speaking women, who were able to fill in the questionnaires, have been included. Therefore, findings may not be generalizable to the whole population. Cultural differences were also not taken into account.

Strength of this study is the fact that ECV was performed in one obstetric department by two trained obstetricians. All data were prospectively recorded. EDS-scores were carefully obtained under supervision of an unbiased research nurse who accompanied the women when completing the EDS-survey. The ratio nulliparous and parous women at the ECV outpatient clinic in this study is in line with the incidence in the general population suggesting that the sample is representative with regard to an important determinant of ECV outcome: parity.

## Conclusions

Psychological factors (fear for ECV and depression EDS-scores) were not related with ECV success rate in this study. Parity, BMI, placental location and engagement of the fetal breech were significantly related with the outcome of ECV. With engagement of the fetal breech being the most important factor associated with a successful ECV.

## Competing interests

The authors declare that they have no competing interests.

## Authors’ contributions

SK, VP and TH were involved in conception and design of the study. EC, ST, MZ, VP analysed the data. EC, SK drafted the first manuscript. EC, MZ, ST, TH, VP, SK contributed to data analysis and interpretation. EC, MZ, ST, TH, VP, SK All authors read and approved the final manuscript.

## Pre-publication history

The pre-publication history for this paper can be accessed here:

http://www.biomedcentral.com/1471-2393/14/101/prepub

## Supplementary Material

Additional file 1: Table S1 Logistic regression of 167 nulliparous women who underwent ECV, outcome successful ECV. Click here for file

Additional file 2: Table S2 Logistic regression of 82 parous women who underwent ECV, outcome successful ECV. Click here for file
