# Supplementary material for: Fear for external cephalic version and depression: predictors of successful external cephalic version for breech presentation at term?
Source: BMC Pregnancy Childbirth. 2014 Mar 12;14:101. doi: 10.1186/1471-2393-14-101 (PMC4007643; doi:10.1186/1471-2393-14-101)
Supplement: Additional file 1: Table S1 — Logistic regression of 167 nulliparous women who underwent ECV, outcome successful ECV. [file 1471-2393-14-101-S1.docx]

***Additional file 1: logistic regression of 167 nulliparous women who underwent ECV, outcome successful ECV***

|  | **Simple logistic regression** | | | **Multiple logistic regression** | | |
| --- | --- | --- | --- | --- | --- | --- |
|  | OR | [95% BI] | P-value | OR | [95% CI] | P-value |
| **Demographic features**  Maternal age (years)  BMI  **Obstetrical features**  Gestational age at ECV  *Type of breech*  Non-Frank  Frank  *Placenta location*  Posterior/ lateral  Anterior  *AFI*  >10  <10  *Tonus of abdominal muscles*  Weak/Normal  Strong  *Tonus of uterus*  Relaxed/normal  Intense  Engagement  Breech above pelvic inlet  Breech in pelvic inlet  Head palpable  Yes  No  EFW (gram)  **Psychosocial features**  Degree of fear before ECV  EDS score before ECV | 1.02  0.90  0.64  2.37  1.00  4.02  1.00  2.58  1.00  1.63  1.00  1.81  1.00  7.16  1.00  2.85  1.00  1.00  0.93  1.01 | [0.95-1.09]  [0.83-0.98]  [0.42-0.99]  [1.19-4.72]  [1.98-8.17]  [1.35-4.92]  [0.77-3.48]  [0.90-3.66]  [3.56-14.40]  [0.76-10.77]  [1.00-1.00]  [0.81-1.07]  [0.93-1.09] | 0.642  **0.016**  **0.047**  **0.014**  **<0.001**  **0.004**  0.205  0.097  **<0.001**  0.122  0.285  0.321  0.874 | 0.92  0.75  1.16  1.00  3.95  1.00  1.98  1.00  6.02  1.00 | [0.84-1.01]  [0.449-1.24]  [0.49-2.76]  [1.75-8.93]  [0.91-4.30]  [2.72-13.30] | 0.080  0.261  0.737  **0.001**  0.086  **<0.001** |

- Bold numbers are statistically significant numbers.
