# Supplementary material for: Fear for external cephalic version and depression: predictors of successful external cephalic version for breech presentation at term?
Source: BMC Pregnancy Childbirth. 2014 Mar 12;14:101. doi: 10.1186/1471-2393-14-101 (PMC4007643; doi:10.1186/1471-2393-14-101)
Supplement: Additional file 2: Table S2 — Logistic regression of 82 parous women who underwent ECV, outcome successful ECV. [file 1471-2393-14-101-S2.docx]

***Additional file 2: logistic regression of 82 parous women who underwent ECV, outcome successful ECV***

|  | **Simple logistic regression** | | | **Multiple logistic regression** | | |
| --- | --- | --- | --- | --- | --- | --- |
|  | OR | [95% BI] | P-value | OR | [95% CI] | P-value |
| **Demographic features**  Maternal age (years)  BMI  **Obstetrical features**  Gestational age at ECV  *Type of breech*  Non-Frank  Frank  *Placenta location*  Posterior/ lateral  Anterior  *AFI*  >10  <10  *Tonus of abdominal muscles*  Weak/Normal  Strong  *Tonus of uterus*  Relaxed/normal  Intense  Engagement  Breech above pelvic inlet  Breech in pelvic inlet  Head palpable  Yes  No  EFW (gram)  **Psychosocial features**  Degree of fear before ECV  EDS score before ECV | 1.13  0.93  1.62  2.90  1.00  1.11  1.00  2.07  3.00  1.00  2.69  1.00  11.43  1.00  2.54  1.00  1.00  0.89  1.08 | [0.98-1.31]  [0.84-1.03]  [0.77-3.38]  [0.86-9.77]  [0.38-3.25]  [0.67-6.19]  [0.61-14.86]  [0.76-9.59]  [3.27-40.01]  [0.39-16.52]  [1.00-1.00]  [0.70-1.13]  [0.93-1.26] | 0.101  0.179  0.200  0.086  0.854  0.195  0.178  0.126  **<0.001**  0.329  0.774  0.347  0.323 | 1.31  1.00  10.46  1.00 | [0.32-5.36]  [2.78-39.31] | 0.707  **0.001** |

- Bold numbers are statistically significant numbers.
